# Supplementary material for: Genetically Proxied Therapeutic Effect of Metformin Use, Blood Pressure, and Hypertension’s Risk: a Drug Target-Based Mendelian Randomization Study
Source: J Cardiovasc Transl Res. 2023 Nov 27;17(3):716–22. doi: 10.1007/s12265-023-10460-z (PMC11219383; doi:10.1007/s12265-023-10460-z)
Supplement: Supplementary file 3 — Supplementary file3 (DOCX 30 KB) [file 12265_2023_10460_MOESM3_ESM.docx]

**STROBE-MR checklist of recommended items to address in reports of Mendelian randomization studies**^1^ ^2^

| **Item No.** | **Section** | **Checklist item** | **Page No.** | **Relevant text from manuscript** |
| --- | --- | --- | --- | --- |
| 1 | **TITLE and ABSTRACT** | Indicate Mendelian randomization (MR) as the study’s design in the title and/or the abstract if that is a main purpose of the study | 2 and 3 | Title: Genetically proxied therapeutic effect of metformin use, blood pressure and hypertension’s risk: A drug-target based Mendelian Randomization study.  In this work, we aim to evaluate the association of the genetically proxied effect of metformin on blood pressure (BP) and hypertension through a drug target-based Mendelian Randomization (MR) analysis. 32 instrumental variables for five metformin targets (i.e. AMP-activated protein kinase (AMPK), growth differentiation factor 15 (GDF15), mitochondrial glycerol 3 (MG3), Mitochondrial complex I (MCI) and glucagon (GCG)) were introduced to the MR analysis on the datasets of hypertension, systolic and diastolic blood pressure (SBP and DBP). |
|  | **INTRODUCTION** |  |  |  |
| 2 | **Background** | Explain the scientific background and rationale for the reported study. What is the exposure? Is a potential causal relationship between exposure and outcome plausible? Justify why MR is a helpful method to address the study question | 3 | A meta-analysis of 26 RCTs containing 4119 patients indicated that metformin could effectively decrease systolic blood pressure (SBP). [5] However, previous findings were inconsistent, with some studies indicating no remarkable effect of metformin on reducing BP. [5] A meta-analysis based on 41 RCTs (3074 patients) reached the opposite conclusion that metformin had no significant effect on BP. [6] Notably, these studies had intrinsic methodologic limitations, including small sample sizes and selection bias. Whether metformin had an effect on BP and hypertension is currently unclear. |
| 3 | **Objectives** | State specific objectives clearly, including pre-specified causal hypotheses (if any). State that MR is a method that, under specific assumptions, intends to estimate causal effects | 3 | Mendelian Randomization (MR) analysis, a complementary and alternative approach to RCTs, is a powerful statistical method that uses the significantly associated single nucleotide polymorphisms (SNPs) as instrumental variables (IVs) to quantify potential causal effects. Drug exerts their effects by regulating the pharmacological targets' expression, and the naturally occurring human genetic variation can serve as a proxy for therapeutic drug targets’ reaction. [7] A recently developed extension to the MR paradigm, i.e. the drug target-based MR study, has been used to find the drug-repurposing candidates for various diseases. [8] The objective of this study was to estimate the causal effect of metformin on hypertension in a larger European population using drug target-based MR. The present findings may potentially offer novel prevention and treatment strategies for hypertension. |
|  | **METHODS** |  |  |  |
| 4 | **Study design and data sources** | Present key elements of the study design early in the article. Consider including a table listing sources of data for all phases of the study. For each data source contributing to the analysis, describe the following: |  |  |
|  | a) | Setting: Describe the study design and the underlying population, if possible. Describe the setting, locations, and relevant dates, including periods of recruitment, exposure, follow-up, and data collection, when available. | 6 | see Figure 1. |
|  | b) | Participants: Give the eligibility criteria, and the sources and methods of selection of participants. Report the sample size, and whether any power or sample size calculations were carried out prior to the main analysis | 6 | see Figure 1. |
|  | c) | Describe measurement, quality control and selection of genetic variants | 3 | see 2.1 part of the Method’s part. |
|  | d) | For each exposure, outcome, and other relevant variables, describe methods of assessment and diagnostic criteria for diseases | 4 | see 2.2 part of the Method’s part. |
|  | e) | Provide details of ethics committee approval and participant informed consent, if relevant | NA | Not applicable. |
| 5 | **Assumptions** | Explicitly state the three core IV assumptions for the main analysis (relevance, independence and exclusion restriction) as well assumptions for any additional or sensitivity analysis | 3 | see 2.1 part of the Method’s part. |
| 6 | **Statistical methods: main analysis** | Describe statistical methods and statistics used |  |  |
|  | a) | Describe how quantitative variables were handled in the analyses (i.e., scale, units, model) | 3 | According to the authors, one standard deviation (SD) unit lowering of the HbA1c equals to 6.75 mmol/mol reduction of HbA1c. |
|  | b) | Describe how genetic variants were handled in the analyses and, if applicable, how their weights were selected | 3 | see 2.1 part of the Method’s part. |
|  | c) | Describe the MR estimator (e.g. two-stage least squares, Wald ratio) and related statistics. Detail the included covariates and, in case of two-sample MR, whether the same covariate set was used for adjustment in the two samples | 4 | In the MR analysis, the inverse-variance weighted (when IVs>2) and Wald ratio (when IVs≤2) approaches were selected to be the major analytical tools. The fixed-effect and random-effect statistical models were employed for the meta-analysis. |
|  | d) | Explain how missing data were addressed | 4 | When the IVs for exposure and its potential proxy SNPs cannot be found in the outcome dataset, they were excluded from further analyses. |
|  | e) | If applicable, indicate how multiple testing was addressed | NA |  |
| 7 | **Assessment of assumptions** | Describe any methods or prior knowledge used to assess the assumptions or justify their validity | NA |  |
| 8 | **Sensitivity analyses and additional analyses** | Describe any sensitivity analyses or additional analyses performed (e.g. comparison of effect estimates from different approaches, independent replication, bias analytic techniques, validation of instruments, simulations) | 5 | The sensitivity analyses on AMPK and MCI suggested that the effects were robust to various MR approaches. (see Table S5-S6) |
| 9 | **Software and pre-registration** |  |  |  |
|  | a) | Name statistical software and package(s), including version and settings used | 4 | All analyses were conducted with R packages MRPRESSO (version 1.0), ieugwasr (version 0.1.5), TwoSampleMR (version 0.5.6), and meta (version 6.0-0). |
|  | b) | State whether the study protocol and details were pre-registered (as well as when and where) | NA |  |
|  | **RESULTS** |  |  |  |
| 10 | **Descriptive data** |  |  |  |
|  | a) | Report the numbers of individuals at each stage of included studies and reasons for exclusion. Consider use of a flow diagram | 6 | see Figure 1. |
|  | b) | Report summary statistics for phenotypic exposure(s), outcome(s), and other relevant variables (e.g. means, SDs, proportions) | 3 and 4 | see 2.1 and 2.2 parts of the method’s part. |
|  | c) | If the data sources include meta-analyses of previous studies, provide the assessments of heterogeneity across these studies | NA |  |
|  | d) | For two-sample MR:  i.  Provide justification of the similarity of the genetic variant-exposure associations between the exposure and outcome samples  ii.  Provide information on the number of individuals who overlap between the exposure and outcome studies | NA |  |
| 11 | **Main results** |  |  |  |
|  | a) | Report the associations between genetic variant and exposure, and between genetic variant and outcome, preferably on an interpretable scale |  | Genetic exposure associations have been reported in the supplementary file ESM Table 2-9 of the paper of Zheng et al.(Zheng et al. Diabetologia. 2022).  The associations between genetic variant and outcome have been summarized in Table S1. |
|  | b) | Report MR estimates of the relationship between exposure and outcome, and the measures of uncertainty from the MR analysis, on an interpretable scale, such as odds ratio or relative risk per SD difference | 8 | see Figure 2. |
|  | c) | If relevant, consider translating estimates of relative risk into absolute risk for a meaningful time period | NA |  |
|  | d) | Consider plots to visualize results (e.g. forest plot, scatterplot of associations between genetic variants and outcome versus between genetic variants and exposure) | 8 | see Figure 2. |
| 12 | **Assessment of assumptions** |  |  |  |
|  | a) | Report the assessment of the validity of the assumptions | 4 | In order to identify the valid IVs, their selection process not only complied with the three key assumptions of MR analysis but also performed additional tests. |
|  | b) | Report any additional statistics (e.g., assessments of heterogeneity across genetic variants, such as *I^2^*, Q statistic or E-value) | 5 | As shown in Table S1-S4, the pleiotropy tests of AMPK- and MCI-specific metformin’s effect on SBP, DBP and hypertension indicated no significant results. We also didn’t detect remarkable heterogeneity results in the AMPK study. However, the test of MCI demonstrated significant heterogeneity results. (See Table S1-S4) |
| 13 | **Sensitivity analyses and additional analyses** |  |  |  |
|  | a) | Report any sensitivity analyses to assess the robustness of the main results to violations of the assumptions | 4 | Other sensitivity methods such as MR Egger, Weighted median, Weighted mode and Simple mode were employed to assess the robustness of the conclusions of the MR analysis with IVs>2. |
|  | b) | Report results from other sensitivity analyses or additional analyses | 5 | The sensitivity analyses on AMPK and MCI suggested that the effects were robust to various MR approaches. (see Table S5-S6) |
|  | c) | Report any assessment of direction of causal relationship (e.g., bidirectional MR) | NA | Not applicable. |
|  | d) | When relevant, report and compare with estimates from non-MR analyses | NA | Not applicable. |
|  | e) | Consider additional plots to visualize results (e.g., leave-one-out analyses) |  |  |
|  | **DISCUSSION** |  |  |  |
| 14 | **Key results** | Summarize key results with reference to study objectives | 9 | Basing on the data from large‐scale GWAS studies in two independent hypertension cohorts of 175648 hypertensive patients, we observed that genetically proxied metformin use leads to a 13% reduction of hypertension risk, which may be owed to its ability to decrease SBP and DBP. Among five drug targets, metformin may exert its anti-blood pressure activities majorly through regulating MG3 and MCI. Collectively, these findings reported a suggestive benefit of metformin in reducing BP and provided novel evidence to guide hypertension prevention. |
| 15 | **Limitations** | Discuss limitations of the study, taking into account the validity of the IV assumptions, other sources of potential bias, and imprecision. Discuss both direction and magnitude of any potential bias and any efforts to address them | 10 | The current work still has limitations. First, though the 5 drug targets may recapitulate the major effect of metformin use, the possibility that some undiscovered targets would greatly affect the metformin response cannot be totally precluded. Second, future studies focusing on the validation of the included DTGs are still needed since the present DTGs derive from the database and systemic literature review and thus lack the direct evidences from biological tests. [9] Third, our study analysed only the data from European participants which may limit the generalizability of the conclusion in other populations. |
| 16 | **Interpretation** |  |  |  |
|  | a) | Meaning: Give a cautious overall interpretation of results in the context of their limitations and in comparison with other studies | 10 | Third, our study analysed only the data from European participants which may limit the generalizability of the conclusion in other populations. |
|  | b) | Mechanism: Discuss underlying biological mechanisms that could drive a potential causal relationship between the investigated exposure and the outcome, and whether the gene-environment equivalence assumption is reasonable. Use causal language carefully, clarifying that IV estimates may provide causal effects only under certain assumptions | 10 | Our findings suggest that MG3 and MCI may be the potential target for the effect of metformin on reducing BP. The MG3 target-associated gene GPD2 encoded the mitochondrial glycerol 3-phosphate dehydrogenase (mGPDH), which is further partly used as a glycerol backbone for lipid molecules. Recent studies have shown that mGPDH was associated with kidney disease and macrophage inflammatory activities. [21, 22] We hypothesized that metformin might reduce BP through these mechanisms. [23] Furthermore, we also found that the inhibition of expression of an MCI-related gene could be associated with the effect of metformin on reducing BP. Intriguingly, the previous report has also confirmed that the inhibition of MCI would diminish hypertension in mice model. [24] |
|  | c) | Clinical relevance: Discuss whether the results have clinical or public policy relevance, and to what extent they inform effect sizes of possible interventions | 10 | We revealed that metformin might decrease BP through the inhibition of MG3 and MCI. These findings implied the repurposing of metformin for cardiovascular diseases. However, randomized trials with large samples are still warranted to provide more robust conclusions. |
| 17 | **Generalizability** | Discuss the generalizability of the study results (a) to other populations, (b) across other exposure periods/timings, and (c) across other levels of exposure | 10 | Third, our study analysed only the data from European participants which may limit the generalizability of the conclusion in other populations. |
|  | **OTHER INFORMATION** |  |  |  |
| 18 | **Funding** | Describe sources of funding and the role of funders in the present study and, if applicable, sources of funding for the databases and original study or studies on which the present study is based | 11 | Funding:  No fund was obtained for this work. |
| 19 | **Data and data sharing** | Provide the data used to perform all analyses or report where and how the data can be accessed, and reference these sources in the article. Provide the statistical code needed to reproduce the results in the article, or report whether the code is publicly accessible and if so, where | 11 | Availability of data and material:  The used GWAS data were publicly available and their origins were described appropriately in the manuscript. The detailed information and codes required to reanalyse the data in this work are available from the corresponding authors upon reasonable request. |
| 20 | **Conflicts of Interest** | All authors should declare all potential conflicts of interest | 11 | Conflict of Interest: The authors declare no conflict of interests. |

This checklist is copyrighted by the Equator Network under the Creative Commons Attribution 3.0 Unported (CC BY 3.0) license.

1. Skrivankova VW, Richmond RC, Woolf BAR, Yarmolinsky J, Davies NM, Swanson SA, et al. Strengthening the Reporting of Observational Studies in Epidemiology using Mendelian Randomization (STROBE-MR) Statement. JAMA. 2021;under review.

2. Skrivankova VW, Richmond RC, Woolf BAR, Davies NM, Swanson SA, VanderWeele TJ, et al. Strengthening the Reporting of Observational Studies in Epidemiology using Mendelian Randomisation (STROBE-MR): Explanation and Elaboration. BMJ. 2021;375:n2233.
