# Supplementary material for: Genetically Proxied Therapeutic Effect of Metformin Use, Blood Pressure, and Hypertension’s Risk: a Drug Target-Based Mendelian Randomization Study
Source: J Cardiovasc Transl Res. 2023 Nov 27;17(3):716–22. doi: 10.1007/s12265-023-10460-z (PMC11219383; doi:10.1007/s12265-023-10460-z)
Supplement: Supplementary file 4 — Supplementary file4 (DOCX 12 KB) [file 12265_2023_10460_MOESM4_ESM.docx]

Table S2 Heterogeneity tests of AMPK-specific metformin effect on SBP, DBP and hypertension

| Exposure | Outcome | Method | Cochrane's Q | Q_pval |
| --- | --- | --- | --- | --- |
| AMPK-specific metformin effect | SBP | MR Egger | 1.6967493 | 0.192713647 |
| AMPK-specific metformin effect | SBP | Inverse variance weighted | 1.956713442 | 0.375928347 |
| AMPK-specific metformin effect | DBP | MR Egger | 4.471512854 | **0.034464463** |
| AMPK-specific metformin effect | DBP | Inverse variance weighted | 4.509229746 | 0.104913941 |
| AMPK-specific metformin effect | Hypertension cohort 1 | MR Egger | 1.824439366 | 0.176785754 |
| AMPK-specific metformin effect | Hypertension cohort 1 | Inverse variance weighted | 2.935170112 | 0.230481413 |
| AMPK-specific metformin effect | Hypertension cohort 2 | MR Egger | 0.061774621 | 0.803712751 |
| AMPK-specific metformin effect | Hypertension cohort 2 | Inverse variance weighted | 0.148546823 | 0.928417819 |
