# Supplementary material for: Genetically Proxied Therapeutic Effect of Metformin Use, Blood Pressure, and Hypertension’s Risk: a Drug Target-Based Mendelian Randomization Study
Source: J Cardiovasc Transl Res. 2023 Nov 27;17(3):716–22. doi: 10.1007/s12265-023-10460-z (PMC11219383; doi:10.1007/s12265-023-10460-z)
Supplement: Supplementary file 5 — Supplementary file5 (DOCX 12 KB) [file 12265_2023_10460_MOESM5_ESM.docx]

Table S3 Heterogeneity tests of MCI-specific metformin effect on SBP, DBP and hypertension

| Exposure | Outcome | Method | Cochrane's Q | Q_pval |
| --- | --- | --- | --- | --- |
| MCI-specific metformin effect | SBP | MR Egger | 81.07517909 | 2.58E-09 |
| MCI-specific metformin effect | SBP | Inverse variance weighted | 81.55631829 | 4.43E-09 |
| MCI-specific metformin effect | DBP | MR Egger | 85.88026384 | 3.86E-10 |
| MCI-specific metformin effect | DBP | Inverse variance weighted | 85.97366677 | 7.92E-10 |
| MCI-specific metformin effect | Hypertension cohort 1 | MR Egger | 40.56948305 | 0.009268566 |
| MCI-specific metformin effect | Hypertension cohort 1 | Inverse variance weighted | 52.79742035 | 0.000390398 |
| MCI-specific metformin effect | Hypertension cohort 2 | MR Egger | 36.34846954 | 0.037995459 |
| MCI-specific metformin effect | Hypertension cohort 2 | Inverse variance weighted | 36.56709456 | 0.048307036 |
