# Supplementary material for: Genetically Proxied Therapeutic Effect of Metformin Use, Blood Pressure, and Hypertension’s Risk: a Drug Target-Based Mendelian Randomization Study
Source: J Cardiovasc Transl Res. 2023 Nov 27;17(3):716–22. doi: 10.1007/s12265-023-10460-z (PMC11219383; doi:10.1007/s12265-023-10460-z)
Supplement: Supplementary file 6 — Supplementary file6 (DOCX 11 KB) [file 12265_2023_10460_MOESM6_ESM.docx]

Table S4 Pleiotropy tests of MCI-specific metformin effect on SBP, DBP and hypertension

| Exposure | Outcome | Egger_intercept | Standard error | pval |
| --- | --- | --- | --- | --- |
| AMPK-specific metformin effect | SBP | -0.031866264 | 0.081411033 | 0.76248237 |
| AMPK-specific metformin effect | DBP | -0.006952095 | 0.075696362 | 0.941695216 |
| AMPK-specific metformin effect | Hypertension cohort 1 | 0.001936608 | 0.002482001 | 0.578183187 |
| AMPK-specific metformin effect | Hypertension cohort 2 | -0.005835452 | 0.019809987 | 0.817628267 |
