# Supplementary material for: Genetically Proxied Therapeutic Effect of Metformin Use, Blood Pressure, and Hypertension’s Risk: a Drug Target-Based Mendelian Randomization Study
Source: J Cardiovasc Transl Res. 2023 Nov 27;17(3):716–22. doi: 10.1007/s12265-023-10460-z (PMC11219383; doi:10.1007/s12265-023-10460-z)
Supplement: Supplementary file 7 — Supplementary file7 (DOCX 11 KB) [file 12265_2023_10460_MOESM7_ESM.docx]

Table S5 Pleiotropy tests of MCI-specific metformin effect on SBP, DBP and hypertension

| Exposure | Outcome | Egger_intercept | Standard error | pval |
| --- | --- | --- | --- | --- |
| MCI-specific metformin effect | SBP | 0.010775346 | 0.031276981 | 0.734056282 |
| MCI-specific metformin effect | DBP | -0.002727907 | 0.018496136 | 0.884225719 |
| MCI-specific metformin effect | Hypertension cohort 1 | 0.001605423 | 0.000623449 | 0.017272136 |
| MCI-specific metformin effect | Hypertension cohort 2 | -0.00201449 | 0.005416196 | 0.713343684 |
