# Supplementary material for: Genetically Proxied Therapeutic Effect of Metformin Use, Blood Pressure, and Hypertension’s Risk: a Drug Target-Based Mendelian Randomization Study
Source: J Cardiovasc Transl Res. 2023 Nov 27;17(3):716–22. doi: 10.1007/s12265-023-10460-z (PMC11219383; doi:10.1007/s12265-023-10460-z)
Supplement: Supplementary file 8 — Supplementary file8 (DOCX 14 KB) [file 12265_2023_10460_MOESM8_ESM.docx]

Table S6 Sensitivity analyses of AMPK-specific metformin effect on SBP, DBP and hypertension

| Exposure | Outcome | Method | nsnp | Beta | Standard error | pval |
| --- | --- | --- | --- | --- | --- | --- |
| AMPK-specific metformin effect | SBP | MR Egger | 3 | 1.878763526 | 1.917998335 | 0.506578439 |
| AMPK-specific metformin effect | SBP | Weighted median | 3 | 1.087168048 | 6.323122552 | 0.863488385 |
| AMPK-specific metformin effect | SBP | Inverse variance weighted | 3 | 1.196119026 | 0.612804128 | **0.050952666** |
| AMPK-specific metformin effect | SBP | Simple mode | 3 | 1.440161309 | 0.985618899 | 0.281440527 |
| AMPK-specific metformin effect | SBP | Weighted mode | 3 | 1.257660981 | 0.692917001 | 0.211180568 |
| AMPK-specific metformin effect | DBP | MR Egger | 3 | 0.565157924 | 1.784351077 | 0.804727015 |
| AMPK-specific metformin effect | DBP | Weighted median | 3 | 0.327632431 | 1.022115756 | 0.748556458 |
| AMPK-specific metformin effect | DBP | Inverse variance weighted | 3 | 0.41615111 | 0.527394197 | 0.430070905 |
| AMPK-specific metformin effect | DBP | Simple mode | 3 | 0.133472947 | 0.577177889 | 0.838624113 |
| AMPK-specific metformin effect | DBP | Weighted mode | 3 | 0.364960506 | 0.39207527 | 0.45020242 |
| AMPK-specific metformin effect | Hypertension cohort 1 | MR Egger | 3 | 0.012946918 | 0.057950818 | 0.860069318 |
| AMPK-specific metformin effect | Hypertension cohort 1 | Weighted median | 3 | 0.048118706 | 0.032188987 | 0.134945486 |
| AMPK-specific metformin effect | Hypertension cohort 1 | Inverse variance weighted | 3 | 0.054092043 | 0.021554683 | **0.012089321** |
| AMPK-specific metformin effect | Hypertension cohort 1 | Simple mode | 3 | 0.048135806 | 0.027735753 | 0.224784814 |
| AMPK-specific metformin effect | Hypertension cohort 1 | Weighted mode | 3 | 0.048135806 | 0.020513309 | 0.143519134 |
| AMPK-specific metformin effect | Hypertension cohort 2 | MR Egger | 3 | -3.346317501 | 3.13672984 | 0.479426158 |
| AMPK-specific metformin effect | Hypertension cohort 2 | Weighted median | 3 | -1.030354661 | 5.515966585 | 0.851821412 |
| AMPK-specific metformin effect | Hypertension cohort 2 | Inverse variance weighted | 3 | -0.925149146 | 1.247533451 | 0.458340247 |
| AMPK-specific metformin effect | Hypertension cohort 2 | Simple mode | 3 | -0.47258866 | 1.928868197 | 0.829295853 |
| AMPK-specific metformin effect | Hypertension cohort 2 | Weighted mode | 3 | -1.13807591 | 1.304804456 | 0.475058472 |
