# Supplementary material for: Genetically Proxied Therapeutic Effect of Metformin Use, Blood Pressure, and Hypertension’s Risk: a Drug Target-Based Mendelian Randomization Study
Source: J Cardiovasc Transl Res. 2023 Nov 27;17(3):716–22. doi: 10.1007/s12265-023-10460-z (PMC11219383; doi:10.1007/s12265-023-10460-z)
Supplement: Supplementary file 9 — Supplementary file9 (DOCX 15 KB) [file 12265_2023_10460_MOESM9_ESM.docx]

Table S7 Sensitivity analyses of MCI-specific metformin effect on SBP, DBP and hypertension

| Exposure | Outcome | Method | nsnp | Beta | Standard error | pval |
| --- | --- | --- | --- | --- | --- | --- |
| MCI-specific metformin effect | SBP | MR Egger | 22 | 0.799560008 | 0.54449525 | 0.157537097 |
| MCI-specific metformin effect | SBP | Weighted median | 22 | 0.399927191 | 0.223601479 | **0.07368388** |
| MCI-specific metformin effect | SBP | Inverse variance weighted | 22 | 0.958757813 | 0.281891757 | **0.000671015** |
| MCI-specific metformin effect | SBP | Simple mode | 22 | 1.672030663 | 0.589354941 | **0.00987273** |
| MCI-specific metformin effect | SBP | Weighted mode | 22 | 0.472401571 | 0.213278055 | **0.037952365** |
| MCI-specific metformin effect | DBP | MR Egger | 22 | 0.892038112 | 0.321318558 | **0.011654386** |
| MCI-specific metformin effect | DBP | Weighted median | 22 | 0.75138836 | 0.128453871 | **4.93E-09** |
| MCI-specific metformin effect | DBP | Inverse variance weighted | 22 | 0.851825466 | 0.166006586 | **2.88E-07** |
| MCI-specific metformin effect | DBP | Simple mode | 22 | 1.009052681 | 0.279823722 | **0.001659689** |
| MCI-specific metformin effect | DBP | Weighted mode | 22 | 0.745950333 | 0.132694068 | **1.40E-05** |
| MCI-specific metformin effect | Hypertension cohort 1 | MR Egger | 24 | 0.01072501 | 0.010708376 | 0.327448886 |
| MCI-specific metformin effect | Hypertension cohort 1 | Weighted median | 24 | 0.016775581 | 0.006640796 | **0.011532342** |
| MCI-specific metformin effect | Hypertension cohort 1 | Inverse variance weighted | 24 | 0.034164984 | 0.00629275 | **5.66E-08** |
| MCI-specific metformin effect | Hypertension cohort 1 | Simple mode | 24 | 0.047784373 | 0.014647464 | **0.00342719** |
| MCI-specific metformin effect | Hypertension cohort 1 | Weighted mode | 24 | 0.017221716 | 0.00598938 | **0.008546414** |
| MCI-specific metformin effect | Hypertension cohort 2 | MR Egger | 25 | -0.098386649 | 0.600942686 | 0.871381328 |
| MCI-specific metformin effect | Hypertension cohort 2 | Weighted median | 25 | -0.012510136 | 0.409165336 | 0.975608663 |
| MCI-specific metformin effect | Hypertension cohort 2 | Inverse variance weighted | 25 | -0.016001979 | 0.330217903 | 0.961350561 |
| MCI-specific metformin effect | Hypertension cohort 2 | Simple mode | 25 | -0.671332206 | 0.761247968 | 0.386587053 |
| MCI-specific metformin effect | Hypertension cohort 2 | Weighted mode | 25 | -0.008287811 | 0.393490501 | 0.983370101 |
